# Supplementary material for: Deciphering Gut Microbiome Responses upon Microplastic Exposure via Integrating Metagenomics and Activity-Based Metabolomics
Source: Metabolites. 2023 Apr 7;13(4):530. doi: 10.3390/metabo13040530 (PMC10145956; doi:10.3390/metabo13040530)
Supplement: Supplementary file 1 [file metabolites-13-00530-s001.zip › metabolites-2097012-supplementary.pdf]

Supplementary Information

Deciphering gut microbiome responses upon microplastic exposure via integrating metagenomics and activity-based metabolomics

Pengcheng Tu[a, 1], Jingchuan Xue[b, 1], Huixia Niu[a, e], Qiong Tang[c], Zhe Mo[a], Xiaodong Zheng[d], Lizhi Wu[a], Zhijian Chen[a], Yanpeng Cai[b], and Xiaofeng Wang[a, *]

[a] Department of Environmental Health, Zhejiang Provincial Center for Disease Control and Prevention, 3399 Binsheng Road, Hangzhou, 310051 China.
[b] Guangdong Provincial Key Laboratory of Water Quality Improvement and Ecological Restoration for Watersheds, Institute of Environmental and Ecological Engineering, Guangdong University of Technology, Guangzhou, 510006 China.
[c] College of Standardization, China Jiliang University, Hangzhou, 310018 China.
[d] Department of Food Science and Nutrition, Zhejiang University, Hangzhou, 310058 China.
[e] School of Medicine, Ningbo University, Ningbo, 315000 China.

[1] Pengcheng Tu and Jingchuan Xue equally contributed to this work.
* Correspondence: xfwang@cdc.zj.cn

Figure. S1 Metabolic impact of MP exposure. (A) Body weight curve. (B) Total weight gain. (C) Food intake. Organ indices of liver (D), kidney (D), and cecum (F). Student's t-test, $p < 0.05$ was considered statistically significant.

Figure. S2 KEGG enrichment analysis of differential metabolites.

Figure. S3 Wilcoxonrank-sum test on phenotype.

Figure. S4 Alterations in metabolites that are related to ABC transporters.

Figure S1

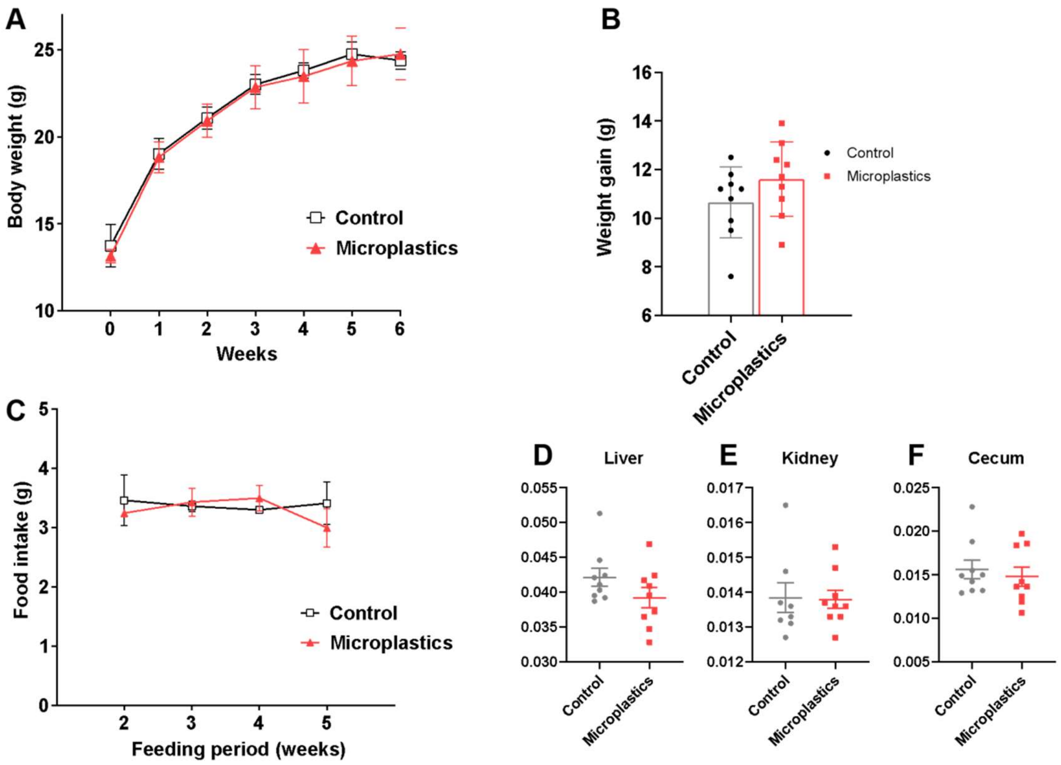
A
Body weight (g)
Weeks
Control
Microplastics
B
Weight gain (g)
Control
Microplastics
C
Food intake (g)
Feeding period (weeks)
Control
Microplastics
D
Liver
E
Kidney
F
Cecum

Figure S2

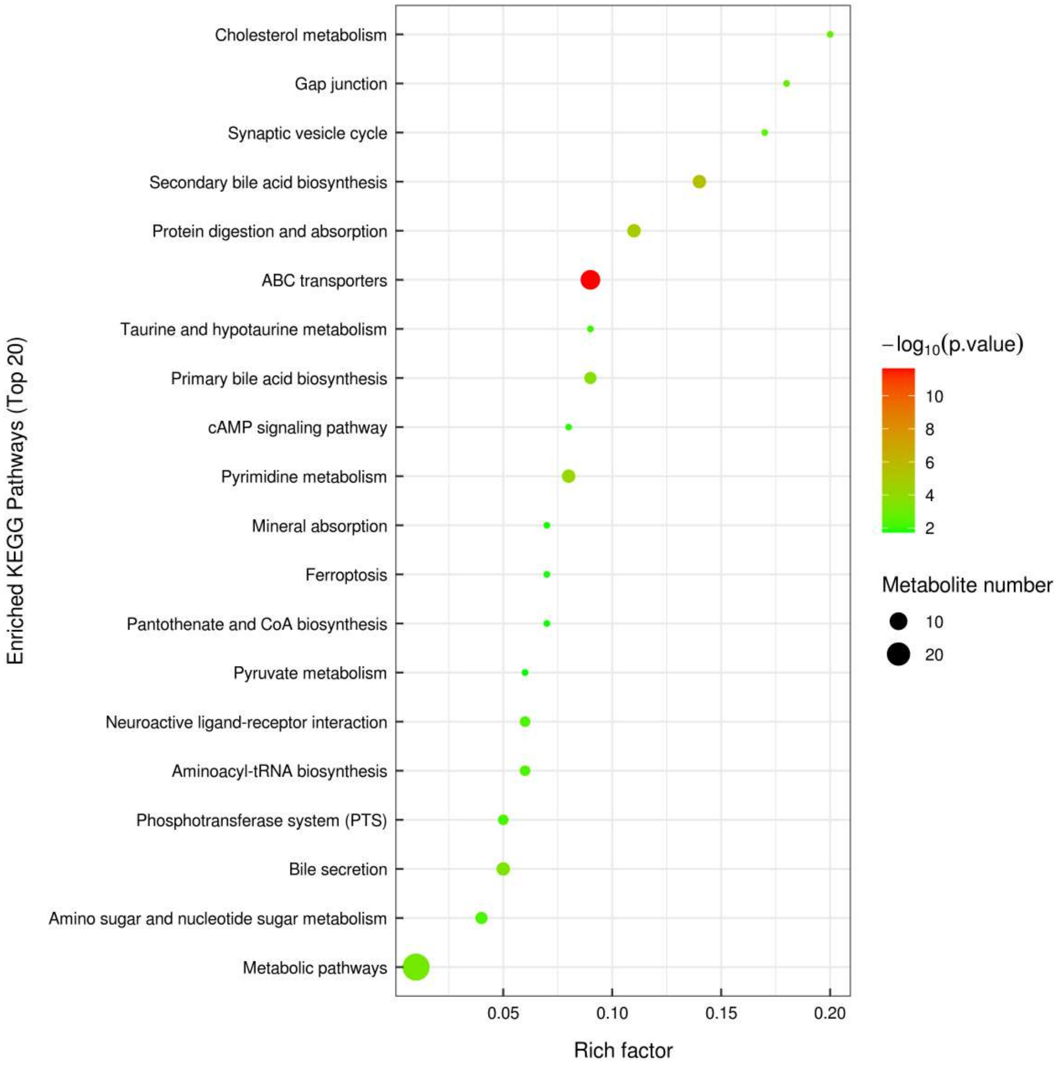

Enriched KEGG Pathways (Top 20)
Cholesterol metabolism
Gap junction
Synaptic vesicle cycle
Secondary bile acid biosynthesis
Protein digestion and absorption
ABC transporters
Taurine and hypotaurine metabolism
Primary bile acid biosynthesis
cAMP signaling pathway
Pyrimidine metabolism
Mineral absorption
Ferroptosis
Pantothenate and CoA biosynthesis
Pyruvate metabolism
Neuroactive ligand-receptor interaction
Aminoacyl-tRNA biosynthesis
Phosphotransferase system (PTS)
Bile secretion
Amino sugar and nucleotide sugar metabolism
Metabolic pathways
0.05
0.10
0.15
0.20
Rich factor
$-\log_{10}$(p.value)
10
8
6
4
2
Metabolite number
10
20

Figure S3

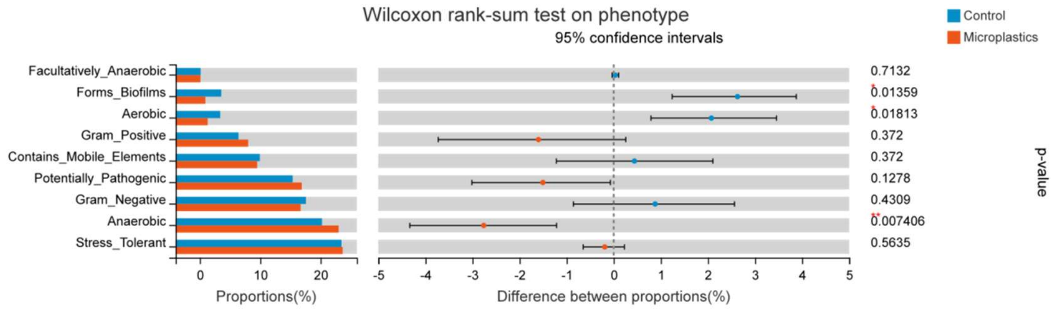

Wilcoxon rank-sum test on phenotype
95% confidence intervals
Control
Microplastics
Facultatively_Anaerobic
Forms_Biofilms
Aerobic
Gram_Positive
Contains_Mobile_Elements
Potentially_Pathogenic
Gram_Negative
Anaerobic
Stress_Tolerant
0.7132
0.01359
0.01813
0.372
0.372
0.1278
0.4309
0.007406
0.5635
p-value
Proportions(%)
Difference between proportions(%)

Figure S4

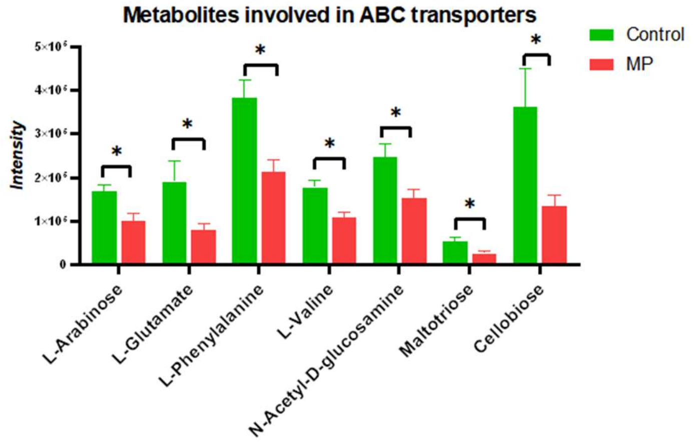

Metabolites involved in ABC transporters
Control
MP
Intensity
5×10⁶
4×10⁶
3×10⁶
2×10⁶
1×10⁶
0
L-Arabinose
L-Glutamate
L-Phenylalanine
L-Valine
N-Acetyl-D-glucosamine
Maltotriose
Cellobiose
